# Artificial Intelligence and Bipolar Disorder: Applications of Machine Learning Models for Diagnosis, Treatment, and Outcome Prediction

**DOI:** 10.31083/AP44494

**Published:** 2025-10-13

**Authors:** Francesco Bartoli, Daniele Cavaleri, Cristina Crocamo

**Affiliations:** ^1^School of Medicine and Surgery, University of Milano-Bicocca, 20900 Monza, Italy

**Keywords:** artificial intelligence, bipolar disorder, machine learning, mental health, predictive learning models

Artificial Intelligence (AI) and its subfields have the potential to transform medical practice and healthcare delivery by addressing the complexities of clinical decision-making [1]. Specifically, machine learning (ML), including deep learning, is a powerful tool that leverages advanced statistical methods and computer-science techniques to analyze large datasets and identify patterns that often elude traditional statistical approaches [2]. ML may be particularly useful in psychiatry, a discipline based on the assessments of diagnostic criteria, by enhancing personalized clinical decision-making [3]. This may be particularly relevant for bipolar disorder (BD); the complex clinical presentation and management of BD [4, 5, 6] can benefit from the potential held by ML. ML techniques may integrate specific information on individual clinical features with other characteristics across different sources of data to make personalized predictions and subsequent treatment decisions [3]. Although still in the earliest stages, research has already begun to show how ML methods are effectively combining heterogeneous data stemming from genetics, electrophysiology, neuroimaging, biomarkers, speech, social media, and mobile health analyses, to improve diagnostic accuracy, identify clinical subtypes of BD, characterize drug-response profiles, and predict illness trajectories.

## 1. Enhancing Diagnostic Accuracy

Differentiating BD from other mental disorders remains a significant clinical challenge [7]. Indeed, BD is often misdiagnosed as major depressive disorder at onset, leading to delays in optimal treatments and poorer clinical outcomes, partly due to the inappropriate use of antidepressant monotherapy [8]. To address this issue, the implementation of ML may support the integration of clinical data to improve diagnostic accuracy. A recent systematic review and meta-analysis [9], based on findings from 18 studies, analyzed 28 ML models to show a pooled sensitivity and specificity of 0.84 and 0.82, respectively, in distinguishing BD from major depressive disorder, demonstrating the strong discriminative potential of these models. Similarly, in another systematic review including 81 studies, ML showed a high degree of accuracy in distinguishing BD from other mental disorders, even though a high risk of publication bias was estimated [10].

## 2. Personalizing Treatment

Beyond diagnosis, ML may be helpful in guiding clinical decision-making in BD. Indeed, predictive models can assist in stratifying patients based on their expected response to different mood stabilizers, antipsychotics, or non-pharmacological interventions, potentially enabling individualized treatment plans. Although research in this area is still limited, recent studies have offered promising preliminary evidence. For instance, interview-based clinical data showed that the response to lithium treatment was predictable, with clinical features such as the characteristics of clinical course, age, age at onset, and sociodemographic features emerging as particularly informative [11]. Consistently, ML models, incorporating polygenic risk scores and clinical factors, appeared effective in identifying patients who are most likely to respond to lithium treatment [12]. However, findings have not been uniformly positive. For instance, ML models applied to electronic health records in the United Kingdom failed to distinguish between lithium and olanzapine responders with BD [13].

## 3. Predicting Clinical Outcomes

The prediction of BD clinical outcomes is likely to be the main strength of ML approaches, as this prediction is critical for an effective management of BD. ML models have demonstrated encouraging performance in this area. A systematic review [14] including 18 studies and over 30,000 participants found that ML models, based on both neuroimaging and clinical data, could predict relapses, hospitalizations, and suicide, with generally acceptable (though heterogenous) performance metrics across studies. That review also identified key clinical predictors of negative outcomes, including early onset, BD-I subtype, comorbid substance use, and circadian-rhythm disruptions, as well as neuroimaging markers involving frontolimbic connectivity and corticostriatal-circuit abnormalities. Moreover, speech markers, identified using both natural language and signal processing from audio data streams of people with BD, have been used to train supervised learning models to assess the feasibility of detection of depressive and manic features [15]. That supports the utility of ML in predicting mood relapses, thereby opening promising perspectives for its integration into digital tools for ecological momentary assessment in psychiatric care [15]. Finally, ML models were also developed and tested to predict mortality. A recent national-register-based cohort study showed good performance in both 2-year and 10-year mortality prediction in both Sweden (*n *= 31,013, followed-up 2006–2021) and Finland (*n *= 13,956, followed-up 1996–2018) [16].

## 4. Challenges and Future Directions

In view of all this, tools containing embedded ML algorithms are likely to markedly enhance decision-making in the clinical management of BD. However, methodological and practical challenges remain. Some validity concerns may be raised regarding the possible relationship between sample size and reported metrics in ML models, that somehow diverge from the expectations set by the theory of learning curves (i.e., performance typically improves or remains stable with increasing sample size) [17]. That suggests that additional factors, potentially including data quality or distribution, may have shaped observed outcomes. Therefore, further studies are needed to validate and replicate findings across different large datasets. In addition, transparent and prespecified analytic protocols are essential to minimize publication and selective-reporting bias. Moreover, the publication of studies on ML models showing low-accuracy results should be encouraged [17]. Finally, possible biases exist, including those related to missing data, misclassification, and measurement error [18]. From a clinical-utility standpoint, the complexity and limited interpretability of several ML models, which appear as “black boxes” providing limited insight into decision-making, may hinder their adoption into routine clinical practice [19]. In response to this, explainable AI (XAI), streamlining of model architecture and the implementing of post-hoc explanations, is gaining increasing attention [20]. Also, inadequate technical infrastructure and unresolved ethical issues may limit the effective adaptation to the clinical contexts in which ML algorithms are intended to be deployed [21]. Risks related to bias in algorithmic decision-making, overreliance on ML outputs, and data-privacy concerns require the adoption of core principles such as beneficence, non-maleficence, autonomy, and justice, especially because of the unique vulnerabilities of psychiatric populations [22, 23].

Notwithstanding the aforementioned problems, ML might be the much sought-after evidence-based, human-centered method that finally gives the clinical characterization of BD a methodological boost and a pragmatic meaning. Future research should prioritize XAI models, promote open and reproducible methods, and foster international collaborations to analyze large, representative datasets that may unlock the full transformative potential of ML in the management of BD. Longitudinal predictive ML models should also integrate clinical, neurobiological, genetic, behavioural, and digital phenotyping data to capture the evolving illness trajectories. Challenging the “one-size-fits-all accuracy-interpretability trade-off”, robust, standardized frameworks for ML validation, including out-of-sample assessment across diverse populations, should be established. These must encompass the entire modeling pipeline (from data preprocessing and feature selection, through model training and evaluation, to deployment), as each stage plays a critical role in ensuring its reliability, generalizability, and safe implementation in psychiatric practice.
